# A Comparison of Three Methods of Mendelian Randomization when the Genetic Instrument, the Risk Factor and the Outcome Are All Binary

**DOI:** 10.1371/journal.pone.0035951

**Published:** 2012-05-09

**Authors:** Philippe Vuistiner, Murielle Bochud, Valentin Rousson

**Affiliations:** University Institute of Social and Preventive Medicine, Centre Hospitalier Universitaire Vaudois and University of Lausanne, Epalinges, Switzerland; University of Bristol, United Kingdom

## Abstract

The method of instrumental variable (referred to as Mendelian randomization when the instrument is a genetic variant) has been initially developed to infer on a causal effect of a risk factor on some outcome of interest in a linear model. Adapting this method to nonlinear models, however, is known to be problematic. In this paper, we consider the simple case when the genetic instrument, the risk factor, and the outcome are all binary. We compare via simulations the usual two-stages estimate of a causal odds-ratio and its adjusted version with a recently proposed estimate in the context of a clinical trial with noncompliance. In contrast to the former two, we confirm that the latter is (under some conditions) a valid estimate of a causal odds-ratio defined in the subpopulation of compliers, and we propose its use in the context of Mendelian randomization. By analogy with a clinical trial with noncompliance, compliers are those individuals for whom the presence/absence of the risk factor *X* is determined by the presence/absence of the genetic variant *Z* (i.e., for whom we would observe *X = Z* whatever the alleles randomly received at conception). We also recall and illustrate the huge variability of instrumental variable estimates when the instrument is weak (i.e., with a low percentage of compliers, as is typically the case with genetic instruments for which this proportion is frequently smaller than 10%) where the inter-quartile range of our simulated estimates was up to 18 times higher compared to a conventional (e.g., intention-to-treat) approach. We thus conclude that the need to find stronger instruments is probably as important as the need to develop a methodology allowing to consistently estimate a causal odds-ratio.

## Introduction

**Figure 1:**
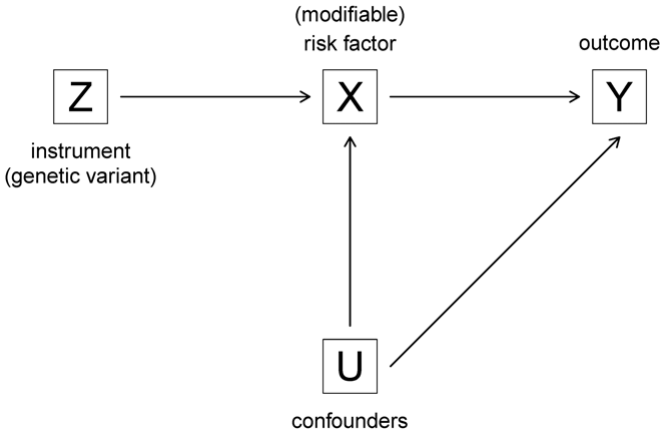
Directed acyclic graph (DAG) representing conditional independencies (by the absence of arrows) between the genetic instrument *Z*, the risk factor *X*, the outcome *Y* and the confounders *U*.

The method of instrumental variable has been introduced nearly one century ago in econometrics [1]. It can be used for estimating a causal effect of a risk factor (predictor, phenotype) *X* on some outcome *Y* in observational studies in epidemiology, where unknown and unmeasured confounding effects *U* are often unavoidable. It can also be used to correct for noncompliance in clinical trials [3]. The method uses an “instrument” *Z* which needs to be (i) correlated with *X*; (ii) independent from *U*; and (iii) conditionally independent from *Y* given *X* and *U* [3], [4], as illustrated in Figure 1. In general, conditions (ii) and (iii) are the problematic ones, since they cannot be verified from the data and should be justified based on subject-matter knowledge. Examples of instruments are the random group assignment in a clinical trial, or a genetic variant associated to the risk factor of interest in an observational study. In the latter case, the method of instrumental variable is often referred to as Mendelian randomization [5].

The method of instrumental variable has been devised to provide a consistent estimate of a causal effect of *X* on *Y* when the relationship is linear, and thus typically applies when the outcome is continuous. It also applies to a binary outcome if the causal effect can be expressed as a risk difference. For a binary outcome, however, a relationship is usually described via an odds-ratio, not a risk difference. Some adaptation of the method of instrumental variable have been proposed to estimate a causal odds-ratio, such as the downloadable qvf function [6] implemented in Stata (Stata Corp, College Station, Texas), or its adjusted version proposed by Nagelkerke et al. [7] and by Palmer et al. [8]. However, it is not yet totally clear in which situations and to which extent these adaptations are valid. In their review, Bochud and Rousson [9] identified 37 observational studies which have used the method of Mendelian randomization between 2004 and 2010, where 23 (i.e. about 60%) considered a binary outcome. They concluded their review stating that “Considering the clear interest for epidemiologists to apply this concept for dichotomous outcomes such as diseases, it would be important, and even urgent, to clarify the issues of the validity of the instrumental variable approach in this context”. Some recent clarification in this regard have been made in Didelez, Meng and Sheehan [10], in Vansteelandt et al. [11] and in Palmer et al. [12].

One conclusion of Palmer et al. [12] was that the above adaptations of the method of instrumental variable should not be used for estimating a causal odds-ratio when *Z*, *X* and *Y* are all binary. However, another estimate of a causal odds-ratio, which also uses an instrumental variable, has recently been proposed by Lui and Chang [13] in the context of a clinical trial with noncompliance. In the present paper, we compare via simulations the usual qvf method and its adjusted version with the method of Lui and Chang [13], confirming that the latter method provides an approximately unbiased estimate of a causal odds-ratio defined in the subpopulation of “compliers”, while illustrating the bias of the former two methods. Thus, we suggest to use the latter method rather than the former two methods in the context of a Mendelian randomization when the genetic instrument *Z*, the risk factor *X* and the outcome *Y* are all binary, and we illustrate its use with an applied example.

The paper is organized as follows. In the Methods section, we recall how the method of instrumental variable can be used to estimate a causal risk difference, and we present the qvf method and its adjusted version for estimating a causal odds-ratio, as well as the method of Lui and Chang [13] which is derived in some details. Our simulations are presented in the Results section, where we also give an example and provide further comparison with other possible estimates of a causal odds-ratio, in particular the logistic structural mean model estimate proposed by Vansteelandt and Goetghebeur [14]. Some concluding remarks take place in the Discussion section.

## Methods

### Estimating a Causal Risk Difference

In this subsection, we recall how it is possible to estimate a causal risk difference using the method of instrumental variable. Let *X* be a risk factor, *Y* an outcome, and *Z* an instrument satisfying the conditions (i), (ii) and (iii) outlined in the Introduction. In this paper, we consider the case where *X*, *Y* and *Z* are all binary (with possible values 0 and 1). Although we shall later switch to the problem of Mendelian randomization, we first consider the case of a randomized clinical trial comparing two treatments with respect to a binary outcome (for which the method of Lui and Chang [13] has been originally derived). There *Z* denotes the random group assignment, while *X* denotes the treatment which is actually taken by the participants. The variables *X* and *Z* may differ for some individuals if noncompliance occurs. In what follows, we consider a sample of *n* individuals, where 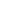 denotes the number of individuals with 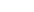, 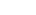 and 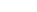 for 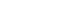. We thus have the situation presented in Table 1.

**Table 1:** Allocation of the *n* individuals of a sample according to *Z*, *X* and *Y*.

	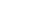		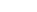	
	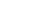	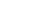	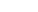	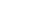
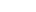	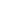	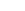	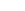	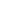
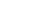	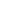	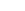	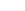	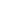

We first review in this subsection how it is possible to estimate the causal effect of the treatment *X* on the outcome *Y* defined via a risk difference. A naive estimate, in what follows the “as-treated” estimate, would simply compare the empirical proportions of 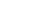 in the groups 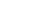 and 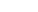 as follows:
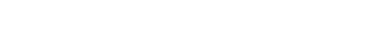



On the other hand, another well-known estimate, the “intention-to-treat” estimate, compares the empirical proportions of 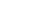 in the groups 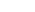 and 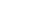 as follows:
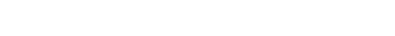



Note that 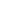 and 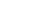 can also be defined as the estimated slopes in a linear regression of *Y* on *X*, respectively of *Y* on *Z*. On the other hand, the “instrumental variable” estimate is a ratio of two slope estimates, from a linear regression of *Y* on *Z* and from a linear regression of *X* on *Z*. The numerator is therefore the intention-to-treat estimate, while the denominator compares the empirical proportion of 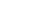 in the groups 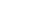 and 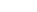. The instrumental variable estimate is thus given by
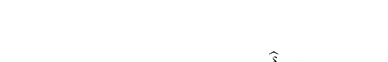



To see which population parameter is hence estimated, we shall distinguish among four categories of individuals, as done in Angrist, Imbens and Rubin [15]. The “compliers” are those individuals for whom a random assignment 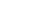 would imply 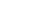 and a random assignment 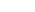 would imply 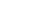. Non-compliers include the “always-takers” (for whom 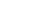 whatever the value of *Z*), the “never-takers” (for whom 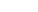 whatever the value of *Z*) and the “defiers” (for whom 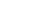 would imply 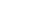 and 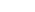 would imply 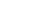). Given the data of a clinical trial, however, it is not possible to tell which of these four categories an individual belongs to, since one cannot infer from the data what he/she would have done if he/she would have been assigned to the other group. In the absence of noncompliance, the three above estimates are identical. When noncompliance occurs, however, these estimates usually differ from each other and converge (as sample size increases) towards different population parameters. Let 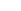, 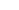, 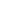 and 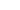 be the proportion of compliers, always-takers, never-takers and defiers in the target population (such that 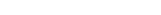). Let 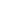, 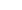, 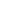 and 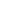 be the proportions of 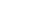 for compliers, always-takers, never-takers and defiers in the group 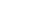, and similarly let 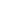, 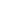, 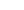 and 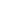 be the proportions of 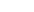 in the group 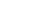. Finally, let 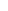 be the proportion of individuals with 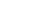 (e.g., 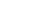 in a clinical trial comparing two groups of equal size). Using condition (ii) in the Introduction section, one can easily derive the following results (in a similar spirit as was done e.g. in Greenland [2]):

•The estimate 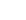 converges towards population parameter

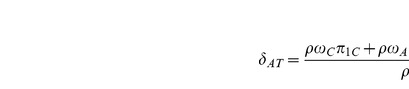
We here compare a group formed of compliers, always-takers and defiers with a group formed of compliers, never-takers and defiers. Since always-takers may have quite different characteristics than never-takers, it is not obvious to provide this parameter with any useful interpretation.

•The estimate 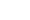 converges towards population parameter

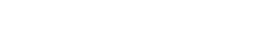



This parameter can be interpreted as the average causal effect of *Z* on *Y* which can be interesting to assess the effect of a public health policy, noncompliance in the sample mimicking the fact that not every person in the target population will strictly follow the official recommendations, for example.

•The estimate 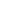 converges towards population parameter

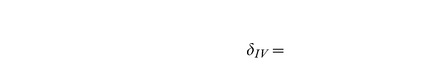



To calculate the denominator, note that the empirical proportion of 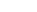 in the group 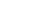 is an estimate of 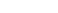, while the empirical proportion of 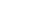 in the group 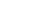 is an estimate of 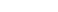, the difference being hence 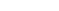.

To get a causal interpretation for the latter estimate, note first that condition (iii) in the Introduction section implies 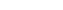 and 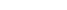 (the outcome *Y* for always-takers and never-takers is not influenced in any respect by the value of *Z*), whereas condition (i) ensures that its denominator does not converge towards zero. One may then make the additional assumption that
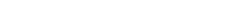



In the terminology of Angrist, Imbens and Rubin [15], (A1) is the “monotonicity assumption”. Using this additional assumption, the estimate 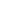 converges thus towards population parameter
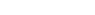



which can be interpreted as the average causal effect of *X* on *Y in the subpopulation of compliers* [15].

In the context of a Mendelian randomization, the instrument *Z* will be a genetic variant associated to a risk factor *X*, and the causal parameter 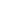 which is estimated using the method of instrumental variable can be interpreted as the risk difference that one would get if one could intervene and change the risk factor *X* from 0 to 1 in the subpopulation of compliers. By analogy with a clinical trial with noncompliance, a complier is an individual for whom the presence/absence of the risk factor *X* is determined by the presence/absence of the genetic variant *Z*, i.e. for whom we would observe 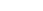 whatever the alleles randomly received at conception. This definition of a complier actually refers to a causal link between *Z* and *X* and we shall also make this assumption in what follows.

### Estimating a Causal Odds-ratio

It is however more common to define the effect of a binary risk factor *X* on a binary outcome *Y* as an odds-ratio rather than a risk difference. Restricting our attention to the subpopulation of compliers, the parameter of interest would thus become
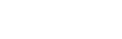



Again, in the context of a Mendelian randomization approach, this causal parameter 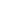 can be interpreted as the odds-ratio which one would get if one could intervene and change *X* from 0 to 1 in the subpopulation of compliers. The naive, or as-treated, estimate of 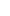 could be expressed as the odds of having 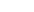 in the group 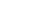 divided by the odds of having 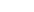 in the group 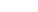, yielding
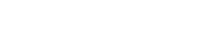
while the intention-to-treat could be expressed as the odds of having 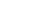 in the group 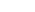 divided by the odds of having 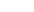 in the group 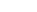, yielding



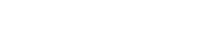



In general, both estimates are not consistent for 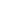 if noncompliance occurs. On the other hand, estimating the parameter 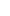 with the classical method of instrumental variable is not obvious. In the qvf function of Stata, one estimates 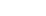 as the ratio of two slope estimates, from a logistic regression of *Y* on *Z* and from a linear regression of *X* on *Z*, yielding
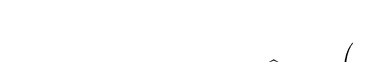



Alternatively, 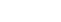 is the estimated slope in a “second stage” logistic regression of *Y* on , where  represents the fitted values calculated from a “first stage” linear regression of *X* on *Z*, that is 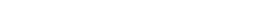 for those individuals with 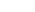 and 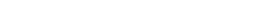 for those individuals with 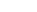. Thus, the 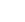 estimate is sometimes referred to as a *two-stages estimate*. Nagelkerke et al. [7], as well as Palmer et al. [8], proposed to improve this estimate by considering in the second stage a logistic regression with *Y* as the response and with two explanatory variables:  and 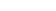 (or equivalently, *X* and *R*). The estimated slope associated to  (respectively to *X*) in this second stage regression is another estimate of 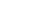, yielding by exponentiation the *adjusted instrumental variable estimate*, in what follows 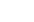. In the econometrics literature, this estimate is known as the “control function estimate”. Note that Palmer et al. [8] considered this estimate with a continuous *X* in the context of Mendelian randomization. Nagelkerke et al. [7] used this estimate 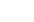 with a binary *X* in the context of a clinical trial with noncompliance, and interpreted it as an estimate of a causal odds-ratio in the subpopulation of the compliers, i.e. as an estimate of 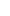.

There is however another method to estimate this causal odds-ratio 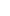, as recently proposed by Lui and Chang [13] and as explained in what follows. While it is not possible to know for each person who is a complier, an always-taker or a never-taker, note that the 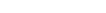 individuals in the first column from Table 1 include compliers and never-takers, the 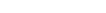 individuals in the second column are always-takers, the 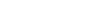 individuals in the third column are never-takers, and the 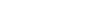 individuals in the last column include compliers and always-takers (recall that we assume no defiers). Since *Z* is an instrumental variable (which is independent from all possible confounding variables), we expect the same proportions of compliers, always-takers and never-takers in both groups (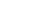 and 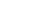). In the group 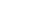, it is hence possible to estimate 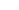 without bias using 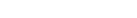. In the group 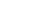, it is possible to estimate 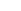 without bias using 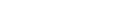. An unbiased estimate of 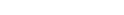 is then obtained as 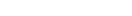. Let 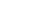 be the proportion of 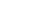 expected in the last column 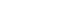 which is equal to
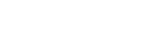



This implies
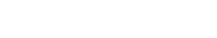



Similarly, let 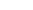 be the proportion of 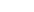 expected in the first column 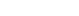 which is equal to
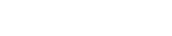



This implies
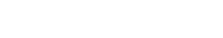



It is then possible to estimate without bias 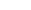 and 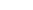 using the data from the last and the first column, respectively yielding 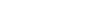 and 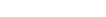. It is also possible to estimate without bias 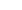 and 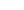 using the data from the second and the third columns, respectively yielding 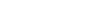 and 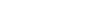. It follows that consistent estimates of 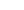 and 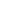 are given by
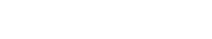
and by



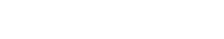
respectively. A consistent estimate of 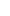 is then given by



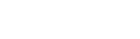
which can also be expressed as



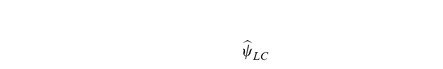



This estimate has been proposed by Lui and Chang [13], without providing all the details about the intermediate estimates 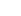, 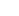, 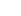, 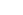 and 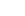 given here, which also provide useful information as illustrated in our example below. In the special case where noncompliance occurs only in one group, this estimate coincides with the estimate proposed by Sommer and Zeger [16]. In a more general context involving a multinomial outcome, Baker [17] showed that the estimates 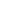 and 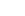 above are the maximum likelihood estimates of 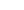 and 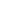 if they are lying between 0 and 1.

## Results

### Simulations

In this subsection, we present the results of simulations which were run to assess the performance of 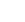, 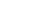 and 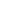 as estimates of the causal parameter 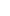 above. Estimates 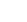 and 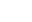 were also included in the comparison. In our simulation design, we considered all possible combinations of the following five factors:

1. *Proportion of compliers* (low, middle, high) 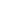 The proportion of compliers was set to 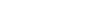 or 0.9, while we took equal proportions of always-takers and never-takers, i.e. 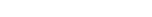.
2. *Baseline prevalence* (small, medium) 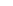 The proportion of 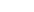 for compliers in the group 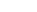 was set to 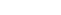 or 0.5.
3. *Odds-ratio* (no effect, medium effect, large effect) 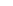 The true odds ratio was set to 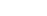 or 9. Thus, we took the proportion of 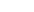 for compliers in the group 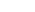 as 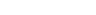 or 0.5 when 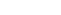, and as 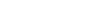 or 0.9 when 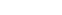.
4. *Confounding effect* (small confounding, high confounding) 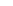 The proportions of 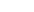 for always-takers was chosen such that the odds was 1.5 or 3 times higher than for compliers, while the proportion of 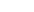 for never-takers was set such that the odds was 1.5 times higher than for compliers. Thus, we took 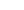 and 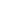 such that 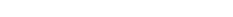 and 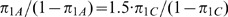, respectively 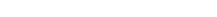. This yielded 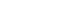 when 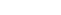, 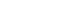 when 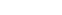, 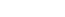 or 0.25 when 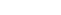, 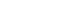 or 0.5 when 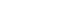, 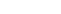 or 0.75 when 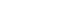, 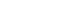 or 0.9 when 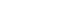 and 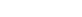 or 27/28 when 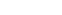.
5. *Total sample size* (small, medium) 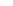 Two sample sizes were used, namely 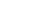 and 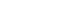, where *n* denotes the total sample size over both groups 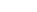 and 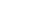, the proportion of individuals with 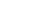 being set to 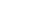 throughout.

For each of these 72 possible combinations of levels, we repeated 2000 simulations. In each replication, the five estimates have been calculated. All simulations were performed using R (version 2.11.1) [18].

We encountered the following technical problems when calculating 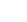. When the true proportion of compliers was low 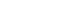, the estimated proportion of compliers 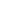 was smaller than zero in 7.7% of the replications when 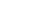 (the problem never happened when 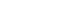). In those cases, we set 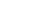 since no effect can be estimated without compliers. Another technical problem was that 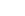 and 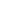 were sometimes outside the range of possible values (0.1). In those cases, values smaller than 0 were set to 0 and values larger than 1 were set to 1, yielding estimated odds-ratios of zero or plus infinity. This situation arose in about 60% of the replications when 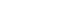 and 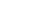. This problem remained in about 15% of the replications when increasing the sample size to 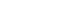, but disappeared when increasing the proportion of compliers to 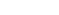.

In each replication, the *F*-statistic from the first stage regression in the instrumental variable approach (the linear regression of *X* on *Z*) was also calculated. According to Stock, Wright and Yogo [19], a value of 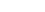 suggests a weak instrument, for which the validity of the inference is not guaranteed. For 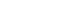 and 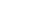, this happened in 95% of the replications. Since the method of instrumental variable is not valid in that setting, we shall not present those results in what follows, leaving us with 60 combinations of levels (this also removed most of the technical problems mentioned above). Note that we still had a value of 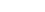 in about 10% of the replications for 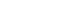 and 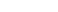, but these replications were kept to avoid a possible selection bias, as explained in Burgess and Thompson [20]. Results are shown in Table 2. To get a robust estimate of the bias and to cope with the estimated odds-ratios of zero or infinity, we report in this table, for each combination of levels and for each estimate, the median of 2000 estimates divided by the true odds-ratio (i.e. 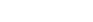). This ratio should be approximately 1 for an unbiased method. In addition, Spearman correlations among the three estimates of main interest 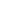, 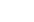 and 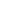 calculated over the 2000 estimates are also reported.

**Table 2:** Summary results of 2000 simulations under various situations for each of the five methods.

design							bias 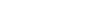					Spearman correlations		
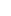	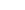	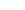	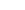	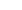	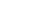	n	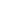	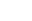	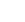	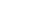	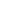	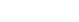	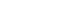	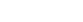
1	0.10	0.10	0.14	0.14	0.50	200	1.006	1.000	1.000	0.999	1.000	1.00	0.97	0.97
3	0.10	0.25	0.14	0.33	0.50	200	1.003	0.531	0.849	0.896	1.033	1.00	0.91	0.91
9	0.10	0.50	0.14	0.60	0.50	200	1.007	0.280	0.707	0.851	1.071	0.99	0.85	0.86
1	0.10	0.10	0.14	0.25	0.50	200	1.533	0.995	0.990	1.003	0.997	1.00	0.96	0.96
3	0.10	0.25	0.14	0.50	0.50	200	1.444	0.494	0.725	0.776	0.996	0.99	0.91	0.91
9	0.10	0.50	0.14	0.75	0.50	200	1.369	0.264	0.639	0.723	1.078	0.99	0.85	0.86
1	0.50	0.50	0.60	0.60	0.50	200	1.008	1.001	1.002	1.004	1.002	1.00	1.00	1.00
3	0.50	0.75	0.60	0.82	0.50	200	0.993	0.585	1.043	1.067	1.020	1.00	0.99	0.99
9	0.50	0.90	0.60	0.93	0.50	200	0.991	0.319	0.940	1.257	1.027	0.98	0.88	0.89
1	0.50	0.50	0.60	0.75	0.50	200	1.364	0.987	0.972	0.966	0.972	1.00	1.00	1.00
3	0.50	0.75	0.60	0.90	0.50	200	1.281	0.602	1.091	1.136	1.029	1.00	0.99	0.99
9	0.50	0.90	0.60	0.96	0.50	200	1.278	0.334	0.999	1.480	1.076	0.97	0.89	0.92
1	0.10	0.10	0.14	0.14	0.90	200	0.990	0.961	0.956	0.955	0.952	0.99	1.00	0.99
3	0.10	0.25	0.14	0.33	0.90	200	1.019	0.870	0.971	0.988	1.025	0.98	0.97	0.97
9	0.10	0.50	0.14	0.60	0.90	200	1.011	0.738	0.908	0.957	1.023	0.91	0.95	0.90
1	0.10	0.10	0.14	0.25	0.90	200	1.137	1.005	1.006	1.009	1.006	0.99	1.00	0.98
3	0.10	0.25	0.14	0.50	0.90	200	1.103	0.824	0.912	0.946	1.007	0.96	0.97	0.95
9	0.10	0.50	0.14	0.75	0.90	200	1.083	0.708	0.879	0.876	1.019	0.82	0.95	0.82
1	0.50	0.50	0.60	0.60	0.90	200	1.003	1.001	1.001	1.001	1.001	0.99	1.00	0.99
3	0.50	0.75	0.60	0.82	0.90	200	1.017	0.903	1.015	1.009	1.009	0.98	1.00	0.97
9	0.50	0.90	0.60	0.93	0.90	200	1.014	0.790	0.978	1.041	1.012	0.96	0.97	0.95
1	0.50	0.50	0.60	0.75	0.90	200	1.069	0.999	0.999	0.996	0.999	0.98	1.00	0.98
3	0.50	0.75	0.60	0.90	0.90	200	1.043	0.912	1.014	1.011	1.005	0.98	1.00	0.98
9	0.50	0.90	0.60	0.96	0.90	200	1.067	0.804	1.003	1.063	1.032	0.95	0.97	0.94
1	0.10	0.10	0.14	0.14	0.10	2000	0.998	1.000	1.002	1.002	1.000	1.00	0.94	0.94
3	0.10	0.25	0.14	0.33	0.10	2000	0.992	0.364	0.792	0.825	1.123	1.00	0.88	0.88
9	0.10	0.50	0.14	0.60	0.10	2000	0.992	0.132	0.603	0.712	1.086	1.00	0.82	0.82
1	0.10	0.10	0.14	0.25	0.10	2000	1.908	1.001	1.007	1.010	1.000	1.00	0.93	0.93
3	0.10	0.25	0.14	0.50	0.10	2000	1.890	0.358	0.671	0.678	1.000	1.00	0.90	0.90
9	0.10	0.50	0.14	0.75	0.10	2000	1.821	0.131	0.572	0.570	1.185	1.00	0.79	0.79
1	0.50	0.50	0.60	0.60	0.10	2000	0.998	0.996	0.958	0.958	0.958	1.00	1.00	1.00
3	0.50	0.75	0.60	0.82	0.10	2000	0.996	0.375	1.069	1.124	1.048	1.00	0.98	0.98
9	0.50	0.90	0.60	0.93	0.10	2000	0.999	0.139	1.019	1.531	1.146	1.00	0.83	0.84
1	0.50	0.50	0.60	0.75	0.10	2000	1.834	1.000	1.002	0.998	1.002	1.00	1.00	1.00
3	0.50	0.75	0.60	0.90	0.10	2000	1.787	0.381	1.249	1.418	1.084	1.00	0.98	0.98
9	0.50	0.90	0.60	0.96	0.10	2000	1.747	0.140	1.099	2.225	1.122	1.00	0.84	0.84
1	0.10	0.10	0.14	0.14	0.50	2000	1.004	1.002	1.005	1.005	1.006	1.00	1.00	1.00
3	0.10	0.25	0.14	0.33	0.50	2000	0.998	0.530	0.843	0.877	1.003	1.00	0.94	0.94
9	0.10	0.50	0.14	0.60	0.50	2000	0.986	0.280	0.705	0.846	1.015	0.99	0.89	0.90
1	0.10	0.10	0.14	0.25	0.50	2000	1.534	1.001	1.003	1.006	1.004	1.00	1.00	1.00
3	0.10	0.25	0.14	0.50	0.50	2000	1.444	0.501	0.754	0.792	1.012	1.00	0.93	0.94
9	0.10	0.50	0.14	0.75	0.50	2000	1.343	0.266	0.636	0.721	1.008	0.99	0.88	0.89
1	0.50	0.50	0.60	0.60	0.50	2000	1.003	1.003	1.007	1.007	1.006	1.00	1.00	1.00
3	0.50	0.75	0.60	0.82	0.50	2000	0.987	0.587	1.035	1.065	0.996	1.00	0.99	0.99
9	0.50	0.90	0.60	0.93	0.50	2000	0.986	0.321	0.928	1.222	1.012	0.98	0.90	0.91
1	0.50	0.50	0.60	0.75	0.50	2000	1.365	1.001	1.001	0.999	1.001	1.00	1.00	1.00
3	0.50	0.75	0.60	0.90	0.50	2000	1.289	0.599	1.078	1.129	0.996	1.00	0.99	0.99
9	0.50	0.90	0.60	0.96	0.50	2000	1.251	0.329	0.979	1.400	1.004	0.98	0.90	0.93
1	0.10	0.10	0.14	0.14	0.90	2000	1.001	1.001	1.001	1.001	1.001	1.00	1.00	1.00
3	0.10	0.25	0.14	0.33	0.90	2000	0.995	0.861	0.955	0.971	0.999	1.00	0.98	0.98
9	0.10	0.50	0.14	0.60	0.90	2000	1.000	0.728	0.897	0.970	1.007	0.99	0.96	0.97
1	0.10	0.10	0.14	0.25	0.90	2000	1.115	1.002	1.002	1.005	1.003	1.00	1.00	1.00
3	0.10	0.25	0.14	0.50	0.90	2000	1.086	0.832	0.922	0.950	1.003	0.99	0.98	0.98
9	0.10	0.50	0.14	0.75	0.90	2000	1.056	0.699	0.858	0.932	1.005	0.98	0.96	0.97
1	0.50	0.50	0.60	0.60	0.90	2000	1.004	1.001	1.001	1.001	1.001	1.00	1.00	1.00
3	0.50	0.75	0.60	0.82	0.90	2000	0.998	0.899	1.004	1.011	1.000	1.00	1.00	1.00
9	0.50	0.90	0.60	0.93	0.90	2000	0.998	0.783	0.973	1.041	1.004	0.99	0.97	0.98
1	0.50	0.50	0.60	0.75	0.90	2000	1.060	0.996	0.995	0.995	0.995	1.00	1.00	1.00
3	0.50	0.75	0.60	0.90	0.90	2000	1.043	0.905	1.009	1.017	0.999	1.00	1.00	1.00
9	0.50	0.90	0.60	0.96	0.90	2000	1.041	0.798	0.991	1.065	1.008	0.99	0.97	0.99

As is well known, the intention-to-treat estimate consistently underestimated the true odds-ratio (even in some situations with 90% of compliers), whereas the as-treated estimate might be biased in both directions, also in cases with no effect. Among the three estimates of main interest, we first notice that 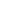 and 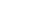 did not differ much, 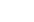 being usually slightly higher than 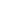 and the correlation between the two estimates being most of the time above 0.98. Both methods were often biased, sometimes downwards and sometimes upwards. The bias was usually larger with higher odds ratios, larger confounding effects and a smaller proportion of compliers. Importantly, the situation did not improve with a larger sample size. By contrast, the bias of the 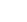 estimate was pretty small, the ratio above being comprised between 0.95 and 1.19 in all considered situations, and the bias would still be smaller by further increasing the sample size.

**Figure 2:**
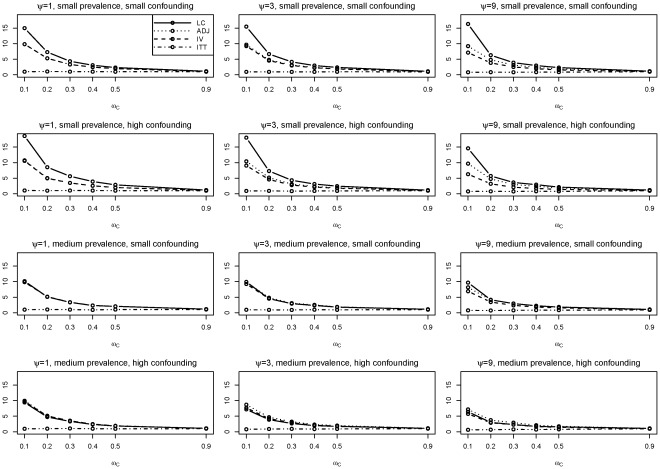
IQR of the log odds-ratios estimated from 2000 simulations under various situations for each of the five methods in function of the proportion of compliers 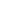. IQR of estimates 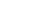 (dashed-dotted line), 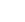 (dashed line), 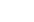 (dotted line) and 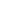 (solid line) have been divided by the IQR of estimate 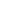. The sample size is 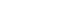.

Besides the bias, we also investigated the variability of the estimates. Figure 2 shows how the inter-quartile range (IQR) calculated from the 2000 estimates of the log odds-ratios depends on the proportion of compliers in the case 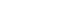 and for different combinations of levels. For this, additional simulations have been carried out with 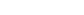 and 0.4 (in addition to 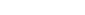 and 0.9). The IQR for the different estimates were divided by the IQR achieved by 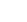, which is the reference method in this figure (would be represented by a horizontal line drawn at the value 1). The three instrumental variable approaches showed a much higher variability than the as-treated and the intention-to-treat estimates, especially when the proportion of compliers was small. For 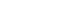, the IQR of 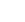 and 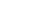 were up to 10 times higher than the IQR of 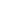, whereas the IQR of 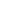 was up to 18 times higher in the case of a small prevalence. For a medium baseline prevalence, the IQR of the three estimates 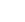, 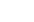 and 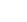 became more comparable with each other. Increasing the level of the odds-ratio or of the confounding effect did not change the results much. The complete results on the IQR for the different estimates are available from the first author upon request.

**Figure 3:**
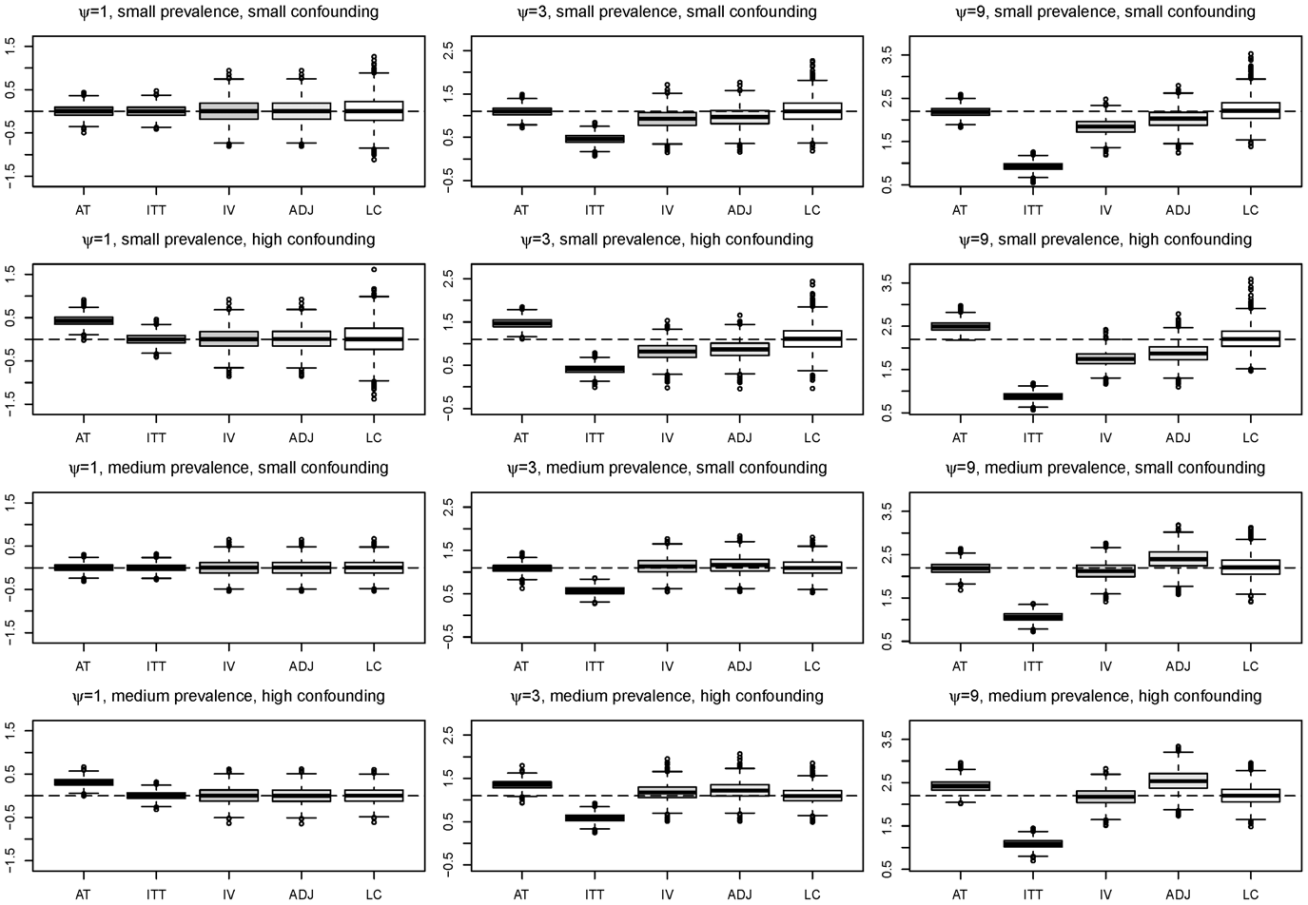
Boxplots of the log odds-ratios estimated from 2000 simulations under various situations for each of the five methods (from left to right: 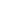, 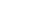, 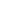, 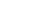, 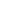). The sample size is 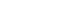 and the proportion of compliers is 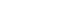. The horizontal dashed line represents the true log odds-ratio.

Figure 3 illustrates both the bias and the variability of the different methods with the boxplots of the 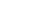 estimates of the log odds-ratios calculated under various settings in the case 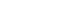 and 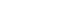. One can retrieve our conclusions above. We also performed simulations using other combinations of levels, e.g., where the proportion of 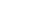 was taken higher for compliers than for always-takers and never-takers, and similar conclusions could be drawn (apart from the direction of the bias for 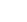, 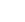 and 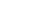).

### Example

In this subsection, we illustrate how the method of Lui and Chang [13] can be used in a context of Mendelian randomization using a partly fictitious example. We consider the effect of alcohol consumption *X* on hypertension *Y*. In what follows, 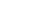 refers to individuals who drink alcohol, and 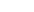 refers to people with hypertension. It is well known that the *aldehyde dehydrogenase 2 (ALDH2)* genotype is strongly associated with alcohol consumption since it encodes an enzyme involved in alcohol metabolism and this relationship might reasonably assumed to be causal [21]. The presence of a protective allele in one of the markers of the *ALDH2* gene has been used as an instrument *Z*, since it is supposed to be responsible for a decrease in alcohol consumption. In what follows, 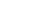 refers to individuals with this protective allele. In this context, a complier is an individual whose phenotype would be determined by his/her genotype (i.e. no alcohol consumption if the protective allele were present (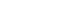), and consumption if the protective allele were absent (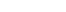)). In contrast, an always-taker is an individual who would drink alcohol, and a never-taker is an individual who would not drink alcohol, whatever his/her genotype. Recall also that we assume no defiers, i.e. there is no one who would drink alcohol if and only if the protective allele were present (i.e. with 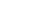 if 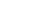 and with 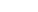 if 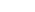), which seems to us tenable (it is in fact not obvious to imagine a subpopulation in which a causal gene would systematically produce the contrary of what it is expected to, although this cannot be verified from the data).

In the study analyzed by Amamoto et al. [22], 51.2% of individuals own the protective allele (i.e. with 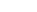). Among persons with 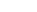, 38.3% suffer from hypertension, whereas this proportion is 48.2% in the group 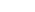. According to the population studied by Yamada et al. [23], the proportion of individuals who drink alcohol in the group 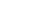 is 90.8% while it is 71.1% in the group 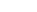. These proportions allow to calculate the margins of a 2×2×2 table summarizing the distribution of 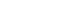. Unfortunately, we did not find comparable data allowing to complete all cells of the table. For the sake of illustration, we complete it by fixing the prevalence of hypertension at 39% in the group with 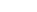 and 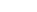, and at 48.5% in the group with 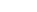 and 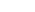. Considering a total sample size of 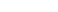 (to match our simulations), this leads to the fictitious data summarized in Table 3.

**Table 3:** Allocation of the 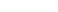 individuals of our example according to *Z*, *X* and *Y*.

	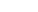		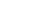	
	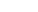	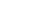	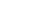	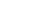
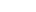	188	444	50	456
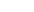	108	284	40	430

Using the formulae given in the Methods section, the proportions of always-takers and never-takers can be estimated as 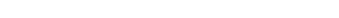 (95%CI: [0.683; 0.739]) and 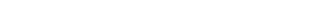 (95%CI: [0.074; 0.110]). This corresponds to an estimated proportion of compliers of 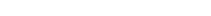 (95%CI: [0.164; 0.230]) (confidence intervals for 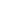, 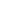 and 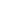 are here obtained by adding and subtracting 1.96 times the standard error of the corresponding estimate, and using the fact that 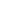 and 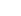 are independent). The proportions of hypertension among always-takers and never-takers are estimated by 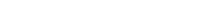 and 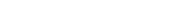. The proportion of hypertension among compliers in the group 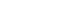 is then estimated as
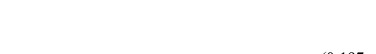
and the proportion of hypertension among compliers in the group 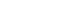 as



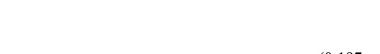



We note in particular that 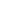 is much higher than 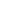, which is informative on the importance of confounding. Finally, the odds-ratio measuring the causal effect of *X* on *Y* for compliers is estimated as
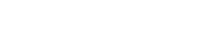
(when keeping all the decimals in the previous calculations). A 95% confidence interval for this odds-ratio calculated as in Lui and Chang [13] yields [2.094; 47.423], which is a wide interval, although it would still indicate a causal odds-ratio which is significantly higher than 2.

Using the other approaches, we obtain 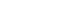 (95%CI: [1.017; 1.604]), 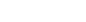 (95%CI: [1.254; 1.790]), 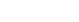 (95%CI: [2.978; 20.317]) and 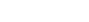 (95%CI: [3.128; 21.887]). The confidence interval associated to 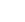 is obtained using the qvf function in Stata, while the confidence interval associated to 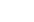 is here computed from 10000 bootstrap replications. Consistent with our simulations, these confidence intervals are somewhat narrower than the confidence interval associated to 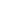, but one cannot here infer anything because of the unknown bias of these methods. We also note that the upper bound of the narrow confidence interval associated to the intention-to-treat estimate, whose bias is known to be in the conservative direction, is still smaller than the lower bound of the confidence interval associated to 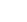.

### Estimating Another Causal Odds-ratio

We have considered so far as target parameter the causal odds-ratio 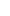 for the subpopulation of compliers. Besides being not identifiable, this subpopulation might admittedly be difficult to apprehend in the context of Mendelian randomization and it will depend on the chosen genetic instrument. Other causal odds-ratios have thus been considered as target parameters in the statistical literature.

In particular, the logistic structural mean model (LSMM) estimate described in Vansteelandt and Goetghebeur [14] has been introduced to estimate a causal odds-ratio 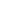 *for a subpopulation of individuals being at risk*, i.e. for whom one would naturally observe 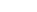. There, the assumption (A1) is replaced by another one:


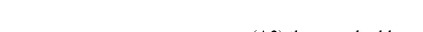
Although the LSMM approach does not rely on (A1), we further assume in what follows that there are no defiers to allow some interesting comparison between the different estimates in that case. According to the terminology employed here, individuals with 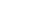 and 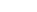 are representative of the always-takers, whereas individuals with 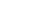 and 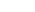 are representative of a subpopulation composed of both the compliers and the always-takers. Thus, using this approach, one is estimating 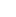 assuming that
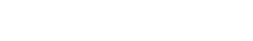
where 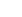 denotes the proportion of 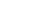 for always-takers that one would get if one could intervene and set 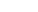, 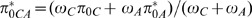 and 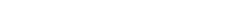 is as in the Methods section. To calculate the LSMM estimate, one may first calculate the estimate 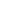 of 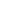 as the value satisfying this assumption, that is



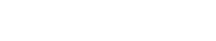
where 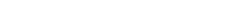 and where 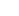, 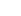, 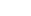, 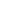, 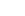 and 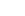 are as in the Methods section. Thus, 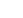 is the plausible solution of the quadratic equation 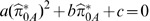, with 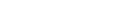, 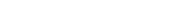 and 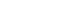, and with 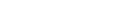, 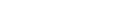 and 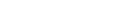. The LSMM estimate 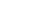 of 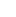 can then be calculated either as



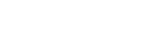
or as



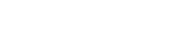



As far as we know, this is an original formulation of the LSMM estimate in the context considered here. One can check that it provides the same result as the equivalent explicit formulation for 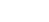 given in the Appendix of Vansteelandt et al. [11]. Applied to our example from the previous subsection, one gets 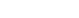 and 
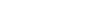
. We note however that the assumption of having the same causal odds-ratio in the subpopulation of always-takers and in the subpopulation of both compliers and always-takers is a very special one (and that its validity will also depend on the chosen genetic instrument). Because of the non-collapsibility of the odds-ratio (when pooling two subpopulations with the same odds-ratio, one does not in general obtain the same odds-ratio, see e.g. Greenland, Robins and Pearl [24]), it does not even imply that the causal odds-ratio is the same for compliers and for always-takers.

Actually, one could alternatively assume that
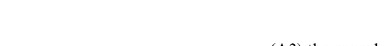
and hence that



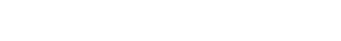
where 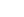 denotes the proportion of 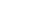 for never-takers that one would get if one could intervene and set 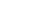. Making this latter assumption, it would then become possible to estimate the causal odds-ratio in the entire population, which we shall denote by 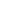. Similarly to the LSMM estimate above, one would first calculate estimates 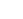 and 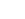 of 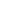 and 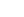 as the values satisfying



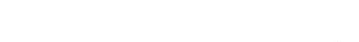
which are given by 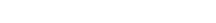 and 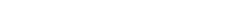. The proportion 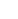 of 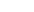 in the entire population that one would get if one could intervene and set 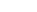 is then estimated as



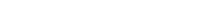
whereas the proportion 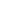 of 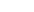 in the whole population that one would get if one could intervene and set 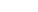 is then estimated as



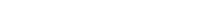



An estimate 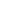 of 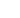 is then simply obtained by
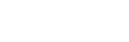



We did not find mention of such an estimate in the literature and it might be interesting to study its statistical properties (although it would rely on both assumptions (A1) and (A3) instead of only either (A1) or (A2)). Applied to our example from the previous subsection, one gets 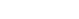, 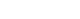, 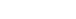, 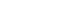 and 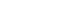. Interestingly, Balke and Pearl [25] have derived bounds for 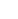 and 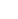 given the (observed) distribution of 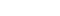, which can then be used to derive bounds for 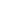. Note also that 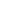 will be necessarily smaller in magnitude than 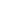 because of the non-collapsibility of the odds-ratio.

## Discussion

In this paper, we have considered the problem of estimating a causal odds-ratio for assessing the effect of a risk factor *X* on an outcome *Y* using Mendelian randomization with a genetic instrument *Z* in the special case where *X*, *Y*and *Z* are all binary. We have confirmed via simulations that the usual adaptations of the method of instrumental variable such as the qvf function of Stata, or the adjusted version considered by Nagelkerke et al. [7] and by Palmer et al. [8] are not valid estimates of the causal odds-ratio in the subpopulation of compliers since a large bias may occur, even with a large sample size. Palmer et al. [12] also recognized that these estimates are not consistent for any causal odds-ratio in this context. By contrast, the method recently proposed by Lui and Chang [13], while being more variable than the two methods above, does not suffer from this bias. While Palmer et al. [12] noted that “estimation of complier causal effects on the odds-ratio scale is more problematic”, it is hence encouraging to have a valid solution in the simple case considered here (i.e. binary *X*, *Y* and *Z*). Further work is needed to estimate whether and how this solution may be extended to more complicated cases.

We have also recalled and illustrated that an instrumental variable approach with a weak instrument, in our context with a low proportion of compliers, might not be very useful because of the huge variability of the estimate. With 10% of compliers, as there are in many examples from the literature, the variability of the estimate of Lui and Chang [13] (measured via the inter-quartile range) can be up to 18 times higher than that of the conventional as-treated or intention-to-treat estimates. With 30% of compliers, the variability can still be up to 5 times higher. In our example, we had about 20% of compliers and the confidence interval obtained for the causal odds-ratio was still rather wide even with a sample size of 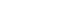. Thus, the need to find stronger instruments is probably as important as the need to develop a methodology allowing to consistently estimate a causal odds-ratio.

Another limitation of the considered approach is that the subpopulation of compliers on which we restrict our attention is in the context of Mendelian randomization “at the least unnatural and a lot harder to grasp” than in the context of a clinical trial with noncompliance, as noted by one anonymous reviewer. While we agree with this statement, the question is whether there really is a viable alternative. If one does not restrict one’s attention to the compliers, one is considering always-takers and never-takers. As it is by definition not possible to observe what would be the outcome of an always-taker if he/she had 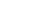, or what would be the outcome of a never-taker if he/she had 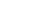, one has to make some speculative assumption in this regard. For example, the assumption which is made when using the logistic structural mean model estimate of Vansteelandt and Goetghebeur [14], for which the target parameter is the causal odds-ratio in a subpopulation of persons being at risk, is that the effect of the risk factor on the outcome is the same in the subpopulation containing the always-takers (and the defiers, if any) and in the subpopulation containing the compliers and the always-takers. While such an assumption might be defendable in a context where the effect is assessed via a risk difference, it seems to us much more questionable in a context where the effect is measured via an odds-ratio (because of the non-collapsibility of the odds-ratio, and unless it is equal to one, it would be quite special to have the same odds-ratio in two subpopulations which partly, but not exactly coincide). This is why we would personally prefer to assume instead that there are no defiers and to use the estimate of Lui and Chang [13] (even if the no defiers assumption is certainly also questionable; we are looking forward to hearing more opinion of geneticists about situations where this assumption might be verified and situations where it might not).

In conclusion, we suggest that the approach of Lui and Chang [13] might be a valuable solution for estimating a causal odds-ratio between a binary risk factor and a binary outcome in the context of a Mendelian randomization with a binary instrument, if we are ready to assume no defiers and despite having to restrict our attention to the compliers. About this latter restriction, we believe that having a valid estimate of the causal effect in a subpopulation of human beings is of scientific interest. Most physiological phenomena have indeed been discovered in a restricted set of people and are usually widely applicable to larger sets of people. That the set of compliers is not an identifiable one should not invalidate this principle.
